# Use of Mid-Upper Arm Circumference by Novel Community Platforms to Detect, Diagnose, and Treat Severe Acute Malnutrition in Children: A Systematic Review

**DOI:** 10.9745/GHSP-D-18-00105

**Published:** 2018-10-03

**Authors:** Jessica Bliss, Natasha Lelijveld, André Briend, Marko Kerac, Mark Manary, Marie McGrath, Zita Weise Prinzo, Susan Shepherd, Noël Marie Zagre, Sophie Woodhead, Saul Guerrero, Amy Mayberry

**Affiliations:** a Center for Global Health, Oregon State University, Corvallis, Oregon, USA.; b Action Against Hunger, London, UK.; c Center for Child Health Research, University of Tampere School of Medicine, Tampere, Finland, and Department of Nutrition, Exercise and Sports, Faculty of Science, University of Copenhagen, Frederiksberg, Denmark.; d London School of Hygiene and Tropical Medicine, London, UK.; e Washington University, St Louis, MO, USA.; f Emergency Nutrition Network, Oxford, UK.; g Nutrition for Health and Development, World Health Organization, Geneva, Switzerland.; h The Alliance for International Medical Action, Dakar, Senegal.; i United Nations Children's Fund, Dakar, Senegal.; j Action Against Hunger, Dakar, Senegal.; k Action Against Hunger, New York, NY, USA.

## Abstract

**Background:**

A stubborn persistence of child severe acute malnutrition (SAM) and continued gaps in program coverage have made identifying methods for expanding detection, diagnosis, and treatment of SAM an urgent public health need. There is growing consensus that making mid-upper arm circumference (MUAC) use more widely accessible among caregivers and community health workers (CHWs) is an important next step in further decentralizing SAM care and increasing program coverage, including the ability of CHWs to treat uncomplicated SAM in community settings.

**Methods:**

We conducted a systematic review to summarize published and operational evidence published since 2000 describing the use of MUAC for detection and diagnosis of SAM in children aged 6–59 months by caregivers and CHWs, and of management of uncomplicated SAM by CHWs, all outside of formal health care settings. We screened 1,072 records, selected 43 records for full-text screening, and identified 22 studies that met our eligibility criteria. We extracted data on a number of items, including study design, strengths, and weaknesses; intervention and control; and key findings and operational lessons. We then synthesized the qualitative findings to inform our conclusions. The issue of treating children classified as SAM based on low weight-for-height, rather than MUAC, at household level, is not addressed in this review.

**Findings:**

We found evidence that caregivers are able to use MUAC to detect SAM in their children with minimal risk and many potential benefits to early case detection and coverage. We also found evidence that CHWs are able to correctly use MUAC for SAM detection and diagnosis and to provide a high quality of care in the treatment of uncomplicated SAM when training, supervision, and motivation are adequate. However, the number of published research studies was small, their geographic scope was narrow, and most described intensive, small-scale interventions; thus, findings are not currently generalizable to public-sector health care systems.

**Conclusions:**

Scaling up the use of MUAC by caregivers and CHWs to detect SAM in household and community settings is a promising step toward improving the coverage of SAM detection, diagnosis, and treatment. Further research on scalability, applicability across a wider range of contexts, coverage impact, and cost is needed. The primary use of MUAC for SAM detection should also be explored where appropriate.

## Abstract (precis)

Limited studies suggest that with robust program inputs caregivers and CHWs can correctly use mid-upper arm circumference to detect severe acute malnutrition (SAM) and that properly trained and supported CHWs can treat uncomplicated SAM in communities.

## INTRODUCTION

Of the approximately 16.4 million children aged 6–59 months worldwide estimated to experience severe acute malnutrition (SAM), roughly 7% to 13% receive treatment each year.^1^^,^^2^ While the growth of community-based management of acute malnutrition (CMAM) programs has considerably increased coverage of treatment for SAM over the past decade, continued gaps in coverage and a persistence of SAM have made identifying strategic methods for expanding access to care, and finding the means to leverage these methods at scale, an urgent public health need.^3^

The use of mid-upper arm circumference (MUAC) by health care providers to detect SAM was inextricably linked to the initial success of CMAM and is likely to remain an accurate, simple, affordable, and acceptable tool to facilitate further scale up of SAM detection and management. In the standard protocol for measuring MUAC to screen for acute malnutrition, a health care provider bends the child's left arm to locate and mark the midpoint. Then the arm is relaxed straight, the MUAC tape is wrapped around the midpoint, and the circumference of the arm is recorded to the nearest 1 millimeter.^1^^,^^2^^,^^4^ The platform for detecting, diagnosing, and treating SAM has typically been within CMAM programs in clinic settings; the merits and limitations of MUAC as an indicator of nutritional and mortality risk in such settings have been well described and debated in the literature.^3^^,^^5^^,^^6^

Despite the word “community” being part of the CMAM acronym, there is seldom a measurement component at the household level. Currently, the standard protocol is being revisited with simpler, alternative protocols in mind, often involving MUAC measurement by community members and caregivers in household and community settings and/or integrating MUAC measurement into other existing platforms, such as part of growth monitoring activities, health campaigns, emergency services, and integrated community case management (iCCM) programs. Expanding the role of community health workers (CHWs) to include detection, diagnosis, and even treatment of uncomplicated SAM is also being explored as an element of decentralizing SAM care. CHWs work in the communities where they reside; we use the term inclusively, referring to both paid and volunteer workers, those working full time, and those working on an ad-hoc basis. CHWs have a decades-long history of successfully diagnosing and treating childhood illness, but their potential for addressing the burden of acute malnutrition remains largely untapped.^7^

The prospect of MUAC-focused management strategies led by caregivers and community members has great potential for enhancing public health impact by facilitating community sensitization and early treatment of affected children, reducing late-stage clinical complications and hospitalizations, and increasing coverage of CMAM programs.^8^ The primary objectives of this systematic review are therefore to summarize the published and operational evidence describing (1) the use of MUAC by caregivers and CHWs in community settings for the detection and diagnosis of SAM, (2) the treatment of SAM by CHWs in community settings, and (3) health platforms where MUAC use and SAM management have been successfully integrated.

## METHODS

This systematic literature review was conducted according to standards set by the Preferred Reporting Items for Systematic Reviews and Meta-Analyses (PRISMA).^9^

Studies were eligible for review if they met the following criteria:

1. The study population included caregivers of children aged 6–59 months with acute malnutrition or those susceptible to acute malnutrition, or CHWs working in the field of acute malnutrition, and
2. The study occurred in a community setting, such as a household or communal space (not in clinics, hospitals, health posts, outreach sites, CMAM sites, or other formal health care settings), and
3. MUAC was used to detect, diagnose, or monitor child anthropometric status, and
4. Outcomes included effectiveness or quality of care provided by CHWs to children with SAM, timeliness of SAM detection or treatment, changes in SAM treatment coverage, operational program descriptions, or other related operational or health outcomes, and
5. Date of publication was 2000 onwards (from the time CMAM programs became operational).

Observational studies, experimental studies, intervention studies, and reviews were all eligible for inclusion. Excluded studies included those that were not available in English, those that did not directly address CHW or caregiver use of MUAC, those that occurred within clinic or hospital settings, and studies of infants younger than 6 months as MUAC is not currently a recommended indicator for acute malnutrition screening in that age group.

Five information sources were used for this review: A database search of peer-reviewed publications was conducted between September 15 and October 15, 2017, using PubMed and Google Scholar; the gray literature was searched on Emergency Nutrition Network (ENN) and Coverage Monitoring Network (CMN) websites between October 15 and October 30, 2017); records from the bibliographies of studies found in our database searches were retrieved between October 15 and October 31, 2017; and lastly, suggestions of relevant sources from experts in the field, including unpublished or operational materials, were received between September 15, 2017, and March 5, 2018.

Our electronic search strategy used the following terms and queries: “community health worker” AND “acute malnutrition” OR “SAM” OR “MUAC”; “reliability” AND “community health worker” AND “anthropometry”; “community screening” AND “malnutrition” AND “child”; “family MUAC”; “MUAC” AND “community diagnosis” OR “community detection”; “acute malnutrition” AND “community detection” OR “community diagnosis”; “MUAC” AND “integration”; “MUAC” AND “health system”; “MUAC” AND “vaccination”; “home-based therapy” AND “SAM”; home-based therapy'” AND “acute malnutrition”.

The study selection process included 4 steps. First, a list of potential studies was compiled via our database search and from expert sources. Second, titles and abstracts were screened based on the eligibility criteria, and third, eligible articles were selected for full-text reading and further screening. The bibliographies of full-text articles were also screened for additional articles that were eligible for inclusion. Finally, articles that met the criteria were submitted for data extraction. For published literature, a standardized form was used for simultaneous data retrieval and data entry of the following items: record reference, objective of study, study design, study population, intervention, control (if any), key findings, operational lessons, study strengths and weaknesses, and comments. For gray literature sources, the reference, organizational source and setting, the objective of the document, and key messages were recorded. Data were extracted by one author and reviewed by second authors. We synthesized the findings of the included studies by summarizing key findings, identifying trends across studies, and noting operational challenges faced during implementation. Disagreements were mutually resolved between all authors. Both the published and the operational materials informed our conclusions, with recognition that individual studies may be biased toward publication of positive results.

## RESULTS

We screened 1,072 records and selected 43 records for full-text screening. We included 22 studies in the review (Figure). A brief summary of each of the 22 studies reviewed is presented in Table 1 (published literature, n=11) and Table 2 (operational materials, n=11). Of the published studies reviewed here, 10 were observational studies describing existing or experimental use of MUAC within communities, and 1 was a randomized control trial evaluating an integrated model for acute malnutrition care. All studies reviewed were conducted in rural settings. Studies included those with paid and volunteer CHWs. The operational materials included 5 reports describing existing community MUAC programs, 3 reports of new tools being developed to facilitate community MUAC use, 1 stakeholder report describing MUAC integration modalities, 1 observational study of community MUAC use, and 1 summary of a randomized control trial to evaluate new acute malnutrition care protocols.

**FIGURE:**
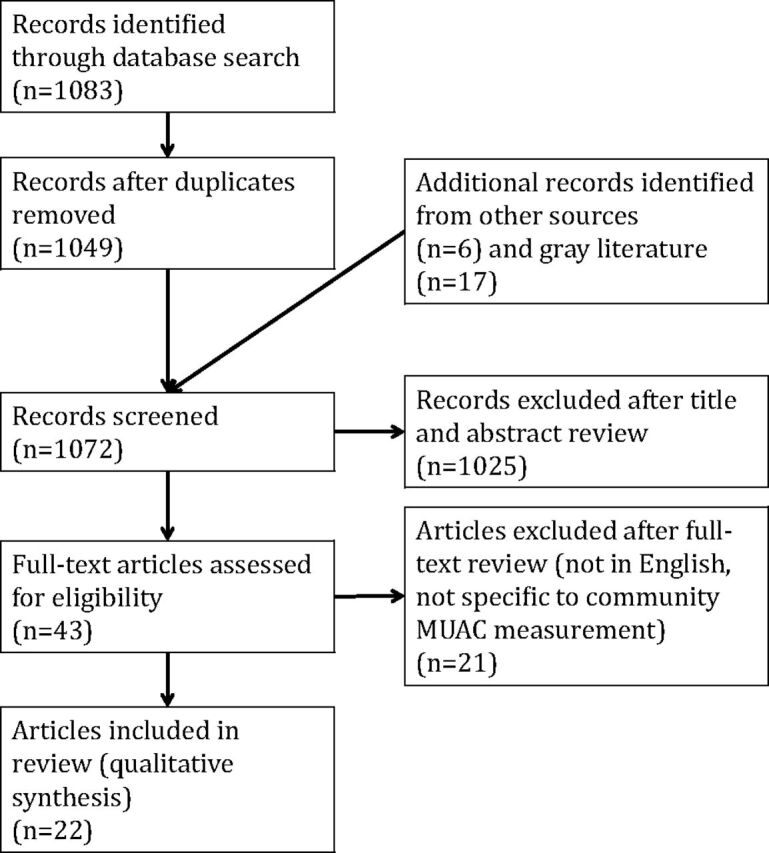
Flow Diagram of Selection Process

**TABLE 1.** Summary of Published Research Studies Included in Review (n=11)

Reference	Objective	Thematic Category and Platform	Design, Training, and Remuneration	Key Findings
Alé et al. 2016^11^	To compare the efficacy and cost-effectiveness of maternal measurement of child MUAC and edema with CHW measurement (Niger)	Caregiver detection, CHW diagnosis (Community platform, rural)	Design: Intervention efficacy study with 2 experimental groups comparing the performance of 12,893 mothers with 36 CHWs Training and remuneration: 30-minute group training plus follow-up individual training for mothers, 6 hours theoretical and 2 hours practical training for CHWs. CHWs were part of established national network and may have been volunteers (payment unknown).	Mothers' MUAC measurements were in agreement with those of health workers more frequently than those made by CHWs (risk ratio 1.88, *P*<.0001). Case detection was earlier in the mothers' group (median MUAC of cases 1.6 mm higher than CHW group), with fewer children requiring inpatient care relative to the CHW group.
Alvarez-Moran et al. 2017^19^	To assess CHW capacity to evaluate, classify, and treat uncomplicated cases of SAM, and to appropriately refer complicated cases, as part of an integrated iCCM package (Mali)	CHW diagnosis and treatment, Integration (iCCM/community platform, rural)	Design: Cross-sectional observational study (no comparison group) of 17 CHWs assessing 125 children Training and remuneration: CHWs had a median of 6 months of job training; no additional training for this study. CHWs were part of Mali's established network and received a salary according to national regulations.	CHWs assessed MUAC correctly in 97% of children, assessed edema correctly in 78%, administered medical treatment correctly in 75% of SAM cases, and managed RUTF supplies correctly in 100% of cases.
Amthor et al. 2009^22^	To describe a rapidly adapted home-based SAM therapy approach in which village health aids diagnosed and treated SAM (MUAC and/or edema) in the context of a food crisis with inadequate health system support (Malawi)	CHW diagnosis and treatment (Emergency community platform, rural)	Design: Retrospective descriptive study of the clinical outcomes of 826 children with SAM who received treatment at home from village health aids Training and remuneration: 5 hours of training plus 5 days job shadowing a nurse. Village health aids were part of an established network; payment unknown.	Recovery rates of children with SAM treated by village health aids were high (94%), without any intervention by medical professionals aside from training. quality of care.
Blackwell et al. 2015^10^	To determine whether minimally trained mothers could identify children with SAM, using either arm and without measuring the specific midpoint (Niger)	Caregiver detection (Community platform, rural)	Design: Nonrandomized non-blinded evaluation study of 2 experimental groups (103 mother-child pairs using simplified protocol and CHWs using standard protocol) Training: Intended to be 5 minutes with each individual, was instead done communally. CHWs were part of a nationally established network and may have been volunteers (unknown).	Mothers' ability to classify GAM and SAM had high sensitivity (>90% of GAM and >73% of SAM cases correctly identified as such) and high specificity (>80% of GAM and >98% of non-cases correctly identified as such). The simplified protocol (either arm and visual ascertainment of midpoint) performed as well as the standard protocol.
Grant et al. 2018^12^	To test the sensitivity of 3 MUAC classification devices when used by caregivers/mothers (Kenya)	Caregiver detection (Community platform, rural)	Design: Prospective nonrandomized clinical diagnostic trial comparing the performance of 3 “Click-MUAC” devices and an MUAC insertion tape across 21 health facilities and 1,040 mother-child pairs Training and remuneration: NA	All devices yielded high sensitivity (>93%) for detecting SAM. Sensitivity for SAM was highest (100%) with the standard MUAC insertion tapes. Specificity was also high for all devices (>96%), with no significant differences observed between the insertion tape and the “Click-MUAC” devices.
Linneman et al. 2007^23^	To assess clinical outcomes of children with acute malnutrition receiving home-based RUTF therapy from community health aids in an operational setting (Malawi)	CHW diagnosis and treatment (Community platform, rural)	Design: Observational study of 3 intervention groups with varying levels of decision-making and SAM treatment authority given to community health aids (12 health centers, >3,000 children with acute malnutrition) Training and remuneration: 1 month plus 4 days job shadowing a nurse. Community health aids were part of an established network; payment unknown.	SAM cases who received treatment from community health aids had the same rate of recovery (90%) as those treated by medical professionals (87%). Note that community health aids appear to have delivered some of the care under supervision in clinic settings.
Maust et al. 2015^27^	To evaluate an integrated MAM/SAM program in terms of coverage, number of children treated, and recovery of children (Sierra Leone)	Integration (Integrated CMAM platform, rural)	Design: Cluster randomized controlled trial with an intervention group (integrated protocol using MUAC for admissions and discharge, RUTF used for MAM and SAM) and a control (standard protocol using W/H Z, RUTF for SAM, and FBFs for MAM) Training and remuneration: NA	Coverage of the integrated program was higher (71% compared with 55% using standard protocol), and recovery rates were comparable (83% vs. 79%).
Nyirandutiye et al. 2011^28^	To evaluate integration of MUAC screening into National Nutrition Week activities (Mali)	Integration (National Nutrition event platform, rural)	Design: Cross-sectional survey of health centers (2) and interviews with health center staff (45), CHWs (17), and caregivers (1543) Training and remuneration: MUAC training was incorporated into event training; CHWs were unpaid volunteers.	Integrating MUAC screening into other activities led to a greater proportion of kids screened (52% of eligible children) than via community screening (5%) or via health center screening (22%), and was viewed as beneficial by caregivers and health care providers. Screening rates were low in clinics, even where staff had been trained in the CMAM protocol.
Puett et al. 2012^20^	To assess the quality of CHW care of uncomplicated SAM cases, including technical competence and acceptability, as part of an iCCM health platform (Bangladesh)	CHW diagnosis and treatment, Integration (iCCM/community platform, rural)	Design: Observational cohort study of 55 CHWs who provided SAM care, and focus group discussions with 29 caregivers whose children received SAM care from CHWs Training and remuneration: 2 days plus monthly refresher trainings. CHWs were part of an established network and received payment.	Trained and supervised CHWs delivered high-quality care to uncomplicated SAM cases; they correctly assessed MUAC and advised caregivers of children with SAM appropriately (90% of cases were managed error-free). Antibiotics correctly administered in 90% of pertinent cases. See also Puett et al. 2013^21^ and Sadler et al. 2011.^25^
Puett et al. 2013^21^	To assess the cost-effectiveness of SAM management (diagnosis and treatment) by CHWs as part of a community nutrition program, compared with inpatient treatment (Bangladesh)	CHW diagnosis and treatment, Integration (iCCM/community platform, rural)	Design: Nonrandomized intervention study of 724 SAM cases treated by CHWs in the community and 633 SAM cases treated as inpatients Training and remuneration: 2 days plus monthly refresher trainings, CHWs were part of an established network and received payment.	CHWs delivered the full spectrum of SAM identification and treatment at a lower overall program cost than inpatient treatment. Supervision was the greatest expense in the CHW group (40% of total, compared with 28% of total budget in inpatient group). See also Puett et al. 2012^20^ and Sadler et al. 2011.^25^
Rogers et al. 2017^24^	To assess the quality of care for uncomplicated SAM by female health workers (Pakistan)	CHW diagnosis and treatment, Integration (iCCM/community platform, rural)	Training: Observational cross-sectional study of 17 female health workers providing care for 61 cases of uncomplicated SAM Training and remuneration: 3 days plus a refresher 3–6 months later. CHWs were part of an existing network and received salaries according to national regulations. They did not receive additional pay for the added SAM care responsibilities.	MUAC and edema were correctly measured for 57% and 88% of children, respectively. 68% of cases received correct medical and nutrition treatment, but only 4% also received key nutritional counseling messages.

Abbreviations: CHW, community health worker; CMAM, community-based management of acute malnutrition; FBF, fortified blended flour; GAM, global acute malnutrition; iCCM, integrated community case management; MAM, moderate acute malnutrition; MUAC, mid-upper arm circumference; NA, not available; RUTF, ready-to-use therapeutic food; SAM, severe acute malnutrition; W/H Z, weight-for-height z score.

**TABLE 2.** Summary of Operational Materials Included in Review (n=11)

Reference	Organizational Source and Setting	Thematic Category	Type of Document	Objective of Document
ACF 2017^42^	Action Against Hunger (DRC, Kenya)	Caregiver detection, CHW diagnosis	Description of program/materials	To describe a simplified, standardized MUAC bracelet under development for testing in the DRC and Kenya.
Bailey 2018^26^	Multiagency (Chad, Kenya, Yemen, Pakistan, Jordan)	Integration	Pilot study (results not yet published)	To summarize the protocol being used by the ComPAS study. The ComPAS study, currently underway as of the writing of this article, aims to integrate the treatment of MAM and uncomplicated SAM by using one product (RUTF) in doses that correspond to growth at each stage of treatment, and using MUAC and edema as the only metrics for admission, monitoring, and discharge.
CMN 2015^34^	Coverage Monitoring Network (no specific setting)	Integration	Advocacy	To advocate for the integration of MUAC into other health and nutrition activities, including vaccination campaigns, well-baby clinics, and water and sanitation programs.
Emary 2017^18^	World Vision (Mauritania)	Caregiver detection	Description of program/materials	To describe qualitative and quantitative tools developed for training and monitoring “Mother-Led MUAC” programs in Mauritania.
Friedman and Wolfheim 2014^35^	Multiagency (no specific setting)	CHW diagnosis and treatment	Description of program/materials	To identify and describe models for how CHWs currently incorporate SAM screening, referrals, and treatment into their work. While there is evidence supporting CHW capacity to conduct all SAM-related activities, there are outstanding questions regarding the conditions that foster success, as well as the optimal mix of iCCM and nutrition-related responsibilities.
ALIMA 2017^15^	ALIMA (Niger, Burkina Faso, Mali, Chad)	Caregiver detection	Description of program/materials	To describe the expansion of “Family MUAC” concepts in Burkina Faso, Chad, Mali, and other locations.
MSF 2017^43^	Médecins Sans Frontières (no specific setting)	Caregiver detection, CHW diagnosis and treatment	Pilot study	To report on lab testing of an alternative MUAC strap for use with adult and child populations. Initial testing of the strap using a standardization process (not on humans, but on differently sized cylinders) showed it to be more accurate and have a higher sensitivity than the standard UNICEF strap. The next step is to test the straps on children in a field setting.
Sadler et al. 2011^25^	Save the Children/Feinstein International Center (Bangladesh)	CHW diagnosis and treatment	Research study	To report outcomes of SAM cases receiving CHW care in Bangladesh (some results also published, see Puett et al. 2012^20^ and Puett et al. 2013^21^). Coverage, weight gain, and recovery were high (89%, 6.7 g/kg/day, and 92%, respectively). The use of multiple pathways to care within the CHW model—use of MUAC, monthly growth monitoring sessions, home visits to sick children, and use of a “watch list” to monitor sick children—facilitated high coverage of screening and diagnosis. See also Puett et al. 2012^20^ and Puett et al. 2013.^21^
Sayadi 2016^17^	CMAM Forum (multiple settings)	Caregiver detection	Description of program/materials	To connect agencies interested in adopting “Mother-Led MUAC” programs (Action Against Hunger, Médecins Sans Frontières, GOAL, Concern, World Vision, International Red Cross, International Medical Corps, and Cooperazione Internazionale).
Sessions 2017^16^	Action Against Hunger (India, Mauritania)	Caregiver detection	Pilot study	To describe 2 pilot studies of the “MUAC Mothers” approach. In India in 2015, 61 caregivers were trained to measure MUAC and given information about how to proceed if they classified their child as having MAM or SAM. Seven months after training, approximately 20 were using the tapes actively; the remaining 41 had misplaced, forgotten how to use the tapes, or not participated in measuring. In Mauritania in 2016, CHWs provided training for more than 6,000 mothers on MUAC use, screening for edema, and what to do if a child got a red, yellow, or green reading.
Tesfai 2015^14^	International Rescue Committee (multiple settings)	CHW diagnosis and treatment	Description of program/materials	To describe tools to enable low-literacy CHWs to diagnose and treat uncomplicated SAM. Piloted tools include use of MUAC-only for admission and monitoring, the use of visual materials (color-coded RUTF dosage charts, scales that indicate RUTF dose, and use of icons to facilitate registration and monitoring), and alignment with iCCM vocabulary and tasks. Field tests have been conducted in Chad, India, Mali, and South Sudan.

Abbreviations: CHW, community health worker; CMAM, community-based management of acute malnutrition; ComPAS, Combined Protocol for Acute Malnutrition Study; DRC, Democratic Republic of the Congo; iCCM, integrated community case management; MAM, moderate acute malnutrition; MUAC, mid-upper arm circumference; NA, not available; RUTF, ready-to-use therapeutic food; SAM, severe acute malnutrition; UNICEF, United Nations Children's Fund.

The studies identified for this review fall into 4 broad, but not mutually exclusive categories:

1. Caregiver detection of SAM using MUAC
2. CHW diagnosis of SAM using MUAC
3. CHW treatment of SAM
4. Integration of MUAC use and/or SAM care into other platforms

Our results and the discussion are organized around these categories.

### Caregiver Detection of SAM Using MUAC in Community Settings

We identified 3 research studies of caregiver detection of SAM using MUAC tapes (Niger^10^^,^^11^) or alternative MUAC devices (Kenya^12^) that indicate that caregiver-focused approaches are reliable and feasible.

Blackwell et al. (2015) found that mothers were capable of using standard MUAC tapes to classify SAM cases with >73% sensitivity and >98% specificity in a pilot study in Niger; this was comparable with CHW performance of 80% and 96%, respectively.^10^ Mothers responded positively to being engaged in monitoring their child's nutritional status, and their comprehension of how MUAC classification corresponded to admission (or exclusion) from SAM treatment programs improved. The simplified MUAC protocol—measurement of either arm, at a midpoint ascertained visually—performed as well as the standard protocol.^10^^,^^13^ In a follow-up study in Niger, Alé et al. (2016) observed that in areas where caregivers were using MUAC tapes to detect SAM in their own households in Niger, the median MUAC of SAM cases admitted to outpatient therapeutic programs was significantly higher than in areas where CHWs were doing the screening.^11^ There were also fewer complicated cases and hospital admissions among mother-referred cases.

Most recently, Grant et al. (2018) compared the performance of 3 prototype “Click-MUAC” devices with an improved MUAC insertion tape (“UniMUAC” tape) among caregivers in Kenya.^12^ The 3 prototypes, which resembled plastic cuffs and tapes, had internal circumferences of 115 mm or 115–125 mm and were hoped to improve case-finding sensitivity over the standard MUAC insertion tapes. Each of the prototypes yielded high sensitivity (>93%) and specificity (>98%), but the UniMUAC tape was superior in both sensitivity (100%) and level of agreement between caregiver, health facility staff, and data collection staff measurements (98%) when screening for SAM.

#### Operational Findings

ALIMA, the nonprofit that runs the Niger-based programs detailed earlier, has expanded its “Family MUAC” programs to Burkina Faso, Chad, and Mali.^14^^,^^15^ Action Against Hunger piloted “MUAC Mothers” programs in India and Mauritania with mixed results. In their India pilot, approximately 30% of mothers trained to use MUAC tapes measured their children in the 7 months following training. In Mauritania, more than 6,000 mothers were trained in both MUAC use and edema detection; outcomes were not available at the time of review.^16^ Several other organizations (Médecins Sans Frontières, GOAL, Concern, World Vision, International Red Cross, International Medical Corps, Cooperazione Internazionale, and Valid International) are in the process of adopting and adapting Family MUAC (also known as “Mother MUAC” and “MUAC Mothers”) programming,^17^ and tools to improve caregiver training and monitoring are in development.^18^

### CHW Detection and Diagnosis in Community Settings

We found evidence supporting high CHW capacity to accurately diagnose SAM using MUAC in 3 studies of 2 interventions in Bangladesh and Mali^19^^–^^21^ Two studies presented mixed evidence from Niger and Pakistan,^11^^,^^13^ and two from Malawi indicated success but did not report specific measured outcomes.^22^^,^^23^

CHWs used standard MUAC approaches to diagnose SAM with a high level of accuracy and reliability, and they found it to be a straightforward tool in work by Alvarez-Moran et al. in Mali (2017).^19^^–^^21^ Alvarez-Moran et al. (2017) found that CHWs correctly assessed MUAC in 97% of children.^19^ In Bangladesh, Puett et al. (2012) observed that CHWs completed MUAC measurements correctly >96% of the time.^20^

We found mixed evidence of CHW MUAC measurement in 2 other studies, one from Niger and one from Pakistan. Alé et al. observed that mothers' MUAC classifications agreed more often with nurses' than those made by CHWs in Niger: mothers' measurements agreed with nurses' 75% of the time, contrasted with only 40% of the time between CHWs and nurses. CHW performance in this case was not described as deficient, despite the discrepancy when compared with caregiver performance.^11^ Rogers et al. (2017) report that MUAC was correctly measured for just 57% of children in their study from Pakistan.^24^ While the authors concluded that CHWs are capable of accurate SAM diagnosis, they speculated that the low rate of correct MUAC measurements was due to operational constraints and low CHW motivation.

### CHW Treatment of SAM in Community Settings

We identified 5 published research studies from Bangladesh, Malawi, and Mali that report consistently successful outcomes of programs or pilot studies of CHW-managed SAM diagnosis and treatment at the household level.^19^^–^^23^ The findings of these studies, which span clinical outcomes of SAM cases, quality of care provided, and cost-effectiveness, indicate that CHWs are capable of providing high-quality, effective care for uncomplicated SAM at a lower cost than inpatient care models, given adequate operational support and supervision.

CHWs provided correct medical care for uncomplicated SAM cases in 75% of cases and managed RUTF supplies correctly for all cases studied by Alvarez-Moran et al. (2017) in Mali.^19^ The majority (80%) of cases were concluded to have received high-quality treatment, defined by Alvarez-Moran as meeting essential indicators across 5 dimensions of care (interface with caregiver, evaluation, classification, treatment, and counseling). Similarly, in Puett's 2012 Bangladesh study, 90% of SAM cases were managed without error; the addition of SAM management to CHWs' regular responsibilities did not appear to affect quality of care or clinical outcomes.^20^ (See also Sadler 2011.^25^) Both Puett et al. (2012) and Alvarez-Moran et al. (2017) determined that high levels of supervision likely contributed to the high quality care they observed in Bangladesh and Mali, respectively.^19^^,^^20^

Amthor et al. (2009) and Linneman et al. (2007) focused on clinical outcomes of SAM cases receiving treatment from CHWs in Malawi; given the high SAM recovery rates (94% and 89%), we presume that the quality of care they received from CHWs was adequate.^22^^,^^23^

We found evidence of unsatisfactory CHW management of SAM in 2 instances, one in the study by Alvarez-Moran et al. in Mali (2017) and in work done by Grant et al. in Pakistan (2018).^19^^,^^24^ Alvarez-Moran et al. (2017) found in Mali that for some tasks, such as administering antibiotics, CHW performance was deficient.^19^ Similarly, in the study by Rogers et al. (2018) in Pakistan, quality of CHW care for SAM was not provided at a consistently high level. While 68% of uncomplicated SAM cases received the correct medical and nutritional care (RUTF, antibiotics, and folic acid), only 4% of cases received the full package of medical and nutritional care and nutritional counseling messages. This low compliance was attributed to operational challenges—namely stock outages—and low CHW motivation owing to lack of extra remuneration.^13^ Nonetheless, and important to note, is the fact that the clinical outcomes of SAM cases in the Pakistan study were noninferior to traditional facility-based models. In other words, failure to deliver the full package of care did not result in low performance or SAM recovery outcomes relative to other care modalities.

#### Operational Findings

There are ongoing efforts to create tools that facilitate SAM diagnosis and treatment by low-literacy CHWs in Chad, India, Mali, and South Sudan. These include the use of visual materials for RUTF dosage and icons to enable reliable case documentation and monitoring.^14^ Simplified dosage protocols that do not depend on measuring child weight, as described in Phase 1 of the Combined Protocol for Acute Malnutrition Study (ComPAS) in Chad, Jordan, Kenya, Pakistan, and Yemen, will also facilitate CHW treatment.^26^ How to safely combine low-literacy tools with the ability to administer antibiotics, assess for danger signs, and refer appropriately when needed requires further exploration.

### Integration of MUAC Use and/or SAM Care Into Other Platforms

Our search yielded 2 published research studies that explicitly described examples of MUAC screening and/or SAM care integration into existing health and/or nutrition platforms. Maust et al. (2015) tested the effectiveness of an integrated treatment program for SAM and moderate acute malnutrition (MAM) in Sierra Leone, which relied on the sole use of MUAC as the indicator used for admissions, monitoring, and discharge, and on RUTF as the sole treatment food. They found the exclusive use of MUAC to be conceptually and logistically simpler, and their results of high coverage (71%) and recovery rates (83% for SAM) are promising.^27^ Other studies (ComPAS) are underway to examine the effectiveness of similar joint protocols in other settings.^26^

Nyirandutiye et al. (2011) reported on the use of MUAC during National Nutrition Week activities in Mali; the event typically has 80% to 90% coverage nationwide and is a promising partner platform for SAM screening and referrals. The difference in screening coverage was substantial: 52% of eligible children (those in the 6–59-month age range) were screened during the event, compared with 22% screened at health centers and 5% screened in the community in the months following the event.^28^ The discrepancies in screening rates suggest that both facility and community-based SAM screening have considerable room for improvement: half of the children with acute malnutrition in their survey had been at a health center within the previous 4 months, but only a quarter of them had been assessed for malnutrition.

#### Operational Findings

The gray literature provides additional support for different models of integration, some of which are already operational and others that are still hypothetical. Friedman and Wolfheim (2014) describe the current range of operational models, 2 of which pertain to SAM screening, referrals, and treatment by CHWs in community settings. In one model, CHWs assess and refer SAM cases as part of iCCM, and in a second model they also provide treatment^29^; we found evidence supporting the success of both models in Bangladesh and Niger in the course of our review.^11^^,^^20^^,^^25^

## DISCUSSION

### Caregiver Detection of SAM Using MUAC in Community Settings

Caregiver-focused models for detecting and classifying SAM using MUAC have potential for increasing coverage and detecting acute malnutrition earlier than standard MUAC protocols. Given the rapid expansion of simplified protocols for caregiver-led pilots and programs, the evidence base is likely to broaden in the next few years. While it is too early to assess the sustainability of caregiver MUAC programs, there is some evidence of high variation in the level of involvement and activity that mothers invest following training. For example, at ALIMA's initial site in Niger, 60% of mothers in the project area have been trained and >70% of CMAM admissions are now referred by mothers.^15^ In contrast, 7 months after an MUAC Mothers training in India, only 30% of mothers reported having ever measured their children.^16^ Understanding how to best motivate and engage caregivers to participate in MUAC measurements and addressing any barriers or stresses created by this responsibility are important questions going forward.

Further simplification of MUAC protocols may hold additional potential in increasing effective community/caregiver MUAC use in other settings. Potential simplifications include embracing “classification” rather than “measurement,” the use of wider color-banded MUAC tapes, the use of either arm for measurements, and/or visually locating the arm midpoint.^10^

### CHW Detection and Diagnosis of SAM Using MUAC in Community Settings

Most of the available evidence supports the ability of CHWs to reliably measure MUAC to the standard necessary for SAM screening. It is worth noting that although MUAC is widely considered a simple indicator for measurement and interpretation, there are examples where the metric was not accepted by CHWs and/or not accurately measured by community workers in the context of growth monitoring and basic anthropometric training^30^^–^^32^ (not included in this review as they did not meet inclusion criteria).

Thus, despite widespread assertions of the simplicity of MUAC, some users—CHWs, nurses, and other health professionals included—find measuring MUAC to be a challenging task. This may partially explain why screening rates and detection of SAM cases remain low even at clinics where staff have been trained in CMAM protocols.^28^ If the findings by Blackwell et al. (2015) hold true, further simplifying the MUAC protocol may address issues of familiarity and understanding of MUAC among health care providers and caregivers alike.^10^

### CHW Treatment of SAM in Community Settings

Most studies reviewed here indicate that CHWs are capable of performing the tasks associated with SAM diagnosis and treatment when supervision, training, and motivation are satisfactory. However, little has been published to describe the effectiveness on child outcomes or coverage relative to standard approaches. One consistent message across studies of CHWs and SAM treatment, both in the published and the gray literature, is the importance of quality training, regular refresher trainings, and high levels of supervision to ensuring sustained CHW motivation, activity, and effectiveness.^19^^,^^24^^,^^25^ The cost of supervision is likely to constitute a large proportion of overall program costs, particularly when implementing a new CHW-led program. Notably, there is evidence that in countries with a well-established CHW network, rapid implementation of CHW-led SAM care in an emergency setting may be highly feasible and effective.^22^

### Integration of MUAC Use and/or SAM Care Into Other Platforms

Several studies indicate that the use of MUAC by caregivers and CHWs is a missing link in the integration of SAM care into other platforms, be it integration with MAM programming;^27^^,^^33^ integration into nutrition or health-focused events such as nutrition weeks, vaccination campaigns, nutrition events, water, sanitation, and hygiene (WASH) programming, or well-baby clinics;^28^^,^^34^ or incorporating MUAC assessment and treatment into existing iCCM programs.^35^ As many of the studies reviewed in this article suggest, and as reported by a study of the integration of SAM care into Niger's national health system, the complexity of acute malnutrition intervention protocols (multiple indicators, treatment types, locations, and steps in care) hinders integration.^36^ Integrating SAM care successfully does not necessarily require integrating every aspect of its treatment, nor is integration a solve-all for inadequate resources or staff.^37^ The optimal mix, or level of mixing, between MUAC use, SAM care, and other activities will likely vary widely depending on context, content, and complementarity of joint activities, among other factors.

### Study Limitations

As with most systematic reviews, this review is subject to publication bias. Published research on MUAC use is likely biased in favor of successful programs; as a result this review may be missing important lessons learned from pilot programs and trials that did not show an apparent positive impact of caregiver or CHW use of MUAC for SAM detection, diagnosis, and/or treatment. Nonetheless, our review did yield work reporting negative outcomes (see Rogers et al. 2017^13^). Most of the studies reviewed here are observational rather than interventional, so inference is limited. Furthermore, most of the published evidence describes intensively delivered, small-scale interventions, and is not readily generalizable to larger scale, public-sector health care systems.

With few exceptions, the studies reviewed here contain little information about the demographic and socioeconomic characteristics of study participants. The absence of this contextual information, the narrow geographic scope of the available studies, and variation in how/whether training and remuneration were delivered limits our ability to generalize findings. Differences in settings, cultures, training protocols, and populations will have implications for the usefulness and acceptability of MUAC.

The issue of treating children classified as SAM based on low weight-for-height, rather than MUAC, at household level, is not addressed in this review. There is evidence that MUAC and weight-for-height do not detect the same children;^38^ however, there is also strong evidence that MUAC is a better predictor of children at high risk of death.^5^^,^^39^ While community studies demonstrate that MUAC is better at predicting mortality, children with low weight-for-height still remain at risk.^5^ Some studies have reassessed MUAC cutoffs in order to better capture children classified as SAM by current weight-for-height criteria.^40^ Others argue that MUAC and weight-for-height identify different children at risk and both should be retained as independent criteria.^41^

We did not address the differences between MUAC and other metrics or implications for the screening, diagnostic, or treatment models reviewed here. Vulnerable children not detected by MUAC alone still need care and should not be overlooked in efforts to scale up and integrate SAM care. Efforts to determine MUAC cutoffs that are sensitive to other metrics—such as low weight-for-height—do exist (see Fiorentino et al. 2016^40^). Determining the impact of CHW treatment programs on children with low weight-for-height will be necessary if an MUAC-focused model is to be rolled out at scale.^8^

## CONCLUSIONS

There is a limited but growing amount of evidence describing the use of MUAC for detection, diagnosis, and treatment of SAM by caregivers and CHWs in community settings. The number of published research studies is small, their geographic scope is narrow, and most describe intensive, small-scale interventions and pilot studies. As such, findings should not yet be extrapolated to other settings. From our review of published research studies, case studies, and operational materials, we can conclude the following:

- Caregivers are able to use MUAC to detect SAM in their children with little apparent risk and many potential benefits to early case detection and coverage.
- CHWs are able to diagnose SAM and provide high-quality treatment with increased coverage, particularly when using simplified protocols and when supported by strong supervision. Without adequate supervision, training, and/or remuneration, the quality of care is likely to suffer. The correct administration of antibiotics by CHWs as well as the ability to detect and refer for danger signs requires further consideration. There is currently only limited evidence on SAM treatment outcomes using this approach, and little consideration of the impact on other child health outcomes under the remit of CHWs.
- While most practitioners consider MUAC relatively simple, it still requires good training and community advocacy in settings where it is unfamiliar. Ongoing research into simplified protocols, modified MUAC tapes, MUAC-based RUTF dosages, and low-literacy treatment tools will all support diagnosis and treatment of SAM by CHWs at household level. The available literature indicates that improvements in coverage are likely when SAM management protocols are simple and the proposed platform for integration is composed of complementary nutrition-related activities.
- In recognizing the benefit of MUAC as a community tool, it is important to recognize that current MUAC criteria do not select for all high-risk children, including low weight-for-height children, and the optimal approach will vary across different contexts. More research is needed to identify different options to identify these high-risk children in the community and ensure successful diagnosis and treatment.

In conclusion, scaling up the use of MUAC to detect SAM in communities is a promising step toward greater coverage and use of existing CMAM services. Given adequate operational support, training, and supervision, the quality of care for self-referred and/or CHW-treated cases is likely to be comparable with current health worker and CMAM models. Further research regarding scalability and applicability of community MUAC use across a wide range of contexts is needed and warranted. In some contexts, the use of MUAC as the primary criterion for detection, diagnosis, and discharge may be appropriate and should be explored further.

## Notes

**Peer Reviewed**

**Funding:** This study was funded by ECHO, grant number ECHO/-WF/BUD/2017/91048.

**First Published Online:** September 5, 2018
